# The benefits of active management of coronary heart disease (CHD) in Wales: how the use of routine collected administrative data can drive efficiency and encourage best practice in condition management.

**DOI:** 10.23889/ijpds.v9i5.1663

**Published:** 2024-09-18

**Authors:** Kendal Smith, Lloyd Evans, Gareth Davies, Ashley Akbari, Rowena Bailey, Richard Palmer, Jonathan Goodfellow, Michael Thomas, Kerryn Lutchman-Singh

**Affiliations:** 1 Welsh Health Specialised Services Committee; 2 NHS Wales; 3 Swansea University; 4 Hywel Dda University Health Board

## Objective and Approach

We analysed the effect on subsequent costs of the setting of the first contact for patients with coronary heart disease in Wales. By categorising health events as proactive or reactive, from the event data source (proactive = primary care (general practice) interactions, elective hospital bed days and outpatient attendances. Reactive = accident and emergency (A&E) attendances and emergency hospital bed days). A standard cost was applied to obtain an annualised cost for each patient. The unit cost for each source was GP £36, bed days £398, outpatient £143, A&E £188.

## Results

We found 42,563 people having CHD events during 2015-2019. Of these, 18,390 had a proactive event as their first event, 23,840 had a reactive event first. The ratio of proactive to reactive events was 1: 3.7 (ratio of costs were £1: £6.30). When the first event was proactive the ratio of events was 1: 0.85 (costs were £1: £1.33). When the first event was reactive the ratio was 1: 4.4 (costs were £1: £33.00).

## Conclusion and Implications

The setting of the first contact appears to be key, with costs reducing by a factor of 14 if the first contact was carried out in a planned environment. Can investment in more active management of a condition reduce overall costs?

The work has highlighted the power of using anonymised population-scale linked individual-level data and indicates the health resource reductions possible from improved managed care of patients.

